# Experiments upon Digestion

**Published:** 1856-07

**Authors:** Francis G. Smith

**Affiliations:** Professor of the Institutes of Medicine in the Medical Department of Pennsylvania College


MEDICAL EXAMINER.

NEW SERIES.—NO. CXXXIX.—JULY, 1856.

### ORIGINAL COMMUNICATIONS.

### EXPERIMENTS UPON DIGESTION.

By Francis G. Smith, M. D.,

Professor of the Institutes of Medicine in the Medical Department of Pennsylvania College.

An opportunity was lately afforded to the writer of examining and experimenting upon Alexis St. Martin, the Canadian, with a fistulous orifice in his stomach; a man whose name is familiar to almost every medical student as the subject of the experiments upon digestion performed by the late Dr. Wm. Beaumont, of the U. S. Army.

It will be remembered that, when quite a lad, St. Martin received the contents of a loaded gun, accidentally discharged. The charge, consisting of powder and duck shot, took effect in his left side, and after tearing away the integuments, muscles, portions of the fifth and sixth ribs and of the left lung and diaphragm, perforated the stomach, creating a wound through which the contents of the latter organ escaped by an orifice large enough to admit the forefinger. This orifice has never closed, although the surrounding wound cicatrised readily.

The man has enjoyed general good health, and has been the father of a large family, whom he has supported, up to the present time, by hard labor.

With one single exception, that of the Esthonian peasant reported by Grunewald and Schroeder, St. Martin is the only instance in which the opportunity of watching the process of digestion, in an otherwise healthy person, has ever been afforded ; the case reported by Circaud in the Jour. de Physiologie, so far as we can learn, never having been experimented upon.*

The experiments performed by Dr. Beaumont are so familiar to every student of physiology, that any analysis of them is uncalled for, even if the limits of this journal permitted it. The object of the present paper is merely to detail the results of recent observations, and compare them with those obtained from other sources.

Several questions relating to the physiological action of the stomach may be regarded as still unsettled ; among these is that relating to the nature of the acid contained in the gastric juice, and the influence of this secretion upon the various alimentary principles as classified by Prout, to wit, saccharine, oleaginous and albuminous food. The present paper will be devoted to the consideration of these points.

It must be premised that the analyses were made upon the fluids obtained from the stomach while digestion was in progress, for that which was withdrawn from the organ while the man was fasting, (that is in the morning, before rising from bed,) was found to be putrescent, although only twenty hours had elapsed after its reception by the writer. The analyses were conducted by the careful hands of Prof. R. E. Rogers, of the University of Pennsylvania, in the presence of the writer, who thus acknowledges the valuable aid received by him.

In every instance, and with all the kinds of food employed, the reaction of the fluid of digestion was distinctly *acid* to litmus paper, while that of the *empty* stomach, as shown by the introduction of test papers through the fistulous orifice, and of the fluid obtained by mechanical irritation, was as distinctly *neutral.* The *temperature* of the stomach, while digestion was in progress, was about 100° to 101° Fahr. When empty, about 98° to 99° Fahr.

The fluid for examination was obtained by placing the man upon his right side, and gently introducing a large sized gum elastic catheter, or a small glass speculum. He was then allowed to turn himself towards the opposite side, when the con-

* Cyc. of Anat. and Phys., Art. Digestion.

tents of the stomach would readily flow out. In no instance was food allowed to remain in the stomach longer than two hours. The mucous membrane of the empty stomach presented a pale pink color, as described by Beaumont, with the surface lubricated by mucus ; when digesting, its color was deepened, and the peristaltic motion could be distinctly seen. During all the experiments St. Martin maintained his usual good health, was in excellent spirits and took his food with appetite.

Previously to the opportunity afforded to Dr. Beaumont by St. Martin's accident, specimens of gastric fluid were obtained by means of sponges attached to strings, as was done by Reaumur and Spallanzani; by exciting vomiting after eating, as recorded by Leipzig; by killing animals, while digestion was going on, as was done by Prout and others; or by establishing fistulous orifices in the stomachs of lower animals, as performed by Blondlot, Lassaigne, Bernard and Barreswill, and others since them.

All these observers, from the earliest to the latest, agree on one point, to wit, the existence of an acid reaction in the fluid of digestion ; but as to the *nature* of that acid, they differ widely, some contending that it is *organic*, others that it is *mineral;* some that is acetic, lactic, or butyric acid, others that the acidity depends upon the presence of hydrochloric acid, or upon the acid biphosphate of lime. The latter theory, advanced by Blondlot, has been disproved by Dumas, Bernard and Melsens, who have shown, that not only the carbonate, but the basic phosphate of lime, are soluble in gastric juice, as are also zinc and iron, with the evolution of hydrogen gas,—properties which a solution of acid phosphate of lime does not possess.*

The analysis of the fluid obtained from St. Martin by Dr. Beaumont in 1833–4, and submitted to Prof. Dunglison, then of the University of Virginia, showed, as the latter states, the presence of "free muriatic and acetic acids, phosphates and muriates, with bases of potassa, soda, magnesia and lime, and an animal matter, soluble in cold water but insoluble in hot." Dr. Dunglison further states, "we distilled the gastric fluid, when the free acid passed over, the salts and animal matter remaining in the

* Phys. Chem., by C. G. Lehmann.

retort. The quantity of chloride of silver thrown down was astonishing."*

Previously to this analysis, in 1824, Dr. Prout had made the same assertion as to the presence of hydrochloric acid, based upon the examination of the contents of the stomachs of rabbits killed while digesting; and Braconnot, in 1835, subsequently to Dunglison, states that he obtained evidences of free hydrochloric acid in gastric juice obtained by sponging the stomachs of animals.†

More recently, Bernard and Barreswill, Pelouse and Thompson, have been led to believe, from their own experimental researches, that *lactic* acid is the agent upon which the characteristic reaction of the gastric juice depends, and attribute the presence of hydrochloric acid in the free state to the decomposition of the alkaline chlorides by the lactic acid at a high heat. Hence, supposing *lactic* acid to be present in the fluid of digestion with the chloride of sodium, the fluid which passes over by distillation will, *at first*, be destitute of hydrochloric acid; but as the liquid becomes more concentrated and the temperature rises, hydrochloric acid will pass over.‡ Lehmann denies the power of hydrochloric acid to decompose the chloride of sodium, but asserts that chloride of calcium is decomposed by lactic acid, even *in vacuo;* and that hence it is not surprising that pure gastric juice should develope vapors *in vacuo*, which, when passed into a solution of nitrate of silver, should form chloride of silver.§

Still more recently, Messrs. Bidder and Schmidt declare, as the result of eighteen corresponding analyses, "that pure gastric juice of carnivora, after eighteen to twenty hours fasting, contained *free hydrochloric acid only*, without a trace of lactic or any other organic acid; while the gastric juice of herbivora contains, with free hydrochloric acid, small quantities of lactic acid, which may, however, be referred to their more amylaceous food."|| Grunnewald's experiments led him to the conclusion

* Experiments and Observations on the Gastric Juice, by Wm. Beaumont, M. D., Surgeon U. S. Army.

† Annales de Chimie, T. 59, p. 348.

‡ Carpenter's Human Physiology, Amer. Edit. p. 109.

§ Phys. Chem. vol. i., p. 93.

|| Cyclopædia of Anat. and Phys., part xlvii., Art. Stomach and Intestines.

that the acid was an organic one; while Schroeder maintains that the fluid obtained by irritating the stomach by peas, owed its reaction to hydrochloric acid.*

Amidst all this conflict of opinion a reconciliation is scarcely to be hoped for; it is suggested, however, that a portion of it, at least, may be owing to the variety of animals experimented upon, and the question may be asked, whether observations made upon the human subject in the healthy condition, should not be relied upon, rather than those derived from experiments performed upon lower animals, in whom the severity of the operation and the emotions necessarily excited thereby, must unavoidably vitiate the results. The difficulty is somewhat relieved by the fact that only *two* acids are involved in the question, and it narrows itself to the decision as to whether they are both present together, or whether one substitutes the other. The following experiments may serve to decide this question.

May 6th, 1856, at 10, A. M.—Two ounces of dry wheat bread were given to St. Martin, which he masticated deliberately and swallowed. At 12½, P. M., the contents of the stomach were removed by Dr. Bunting in the presence of a number of medical gentlemen and students, and carefully preserved for immediate analysis. The reaction was *decidedly* acid, s. g. 1009. Microscopic examination showed large epithelial cells, mucous corpuscles, amorphous granular matter and starch granules, some broken down, others perfect, together with a few cells of cylinder epithelium.

*Experiment* 1.—A portion of the fluid thus obtained, was subjected to distillation. In the early vapor that came over, no trace of acidity could be detected by litmus paper; the distillate was neutral, neither acid nor alkaline, and did not precipitate with nitrate of silver. The distillation being carried further, so as to concentrate the material in the retort and increase its temperature, the distillate was found to become acid, and a portion being added to a solution of nitrate of silver, a faint precipitate, which was soluble in ammonia, took place. (This experiment has been repeated since, with the material discharged from the stomach, *at will*, after a meal of bread. The distillate became *distinctly* acid, but threw down the faintest

* Dissert. Inaug.

precipitate, a mere opalescence, with nitrate of silver. The acid of the distillate gave all the evidence of lactic acid.)

*Ex.* 2.—A portion of the material from the retort being tested with chloride of barium, gave no visible indication of sulphuric acid.

*Ex.* 3.—Another portion of the gastric fluid was heated in a porcelain capsule for the purpose of incineration. The vapor that escaped gave no evidence of acidity, but the residue became increasingly acid in proportion as it became more concentrated.

May 9th. Two ounces of bread moistened with water, were introduced into the stomach, through the fistulous orifice. In an hour and a half the contents were withdrawn. The fluid was very viscid, and presented, as before, a decidedly acid reaction. Some portions of the bread were undissolved, although the greater part had disappeared; sp. gr. not noted. The microscope revealed fewer epithelial cells than in the examination of the previous fluid, some mucous corpuscles and abundance of starch granules, some of which were broken down.

*Ex.* 4.—A portion of the fluid just obtained was decanted from the bread particles and carefully distilled, without presenting any evidence of acidity to litmus in the fumes. The distillate was acid to litmus, and when tested with nitrate of silver, presented a *very faint* indication of the preseuce of hydrochloric acid. The residue in the retort, when somewhat concentrated, gave a deep acid reaction.

*Ex.* 5.—A portion of the same fluid, as in Ex. 4, was gently boiled in a retort; the distillate was acid, and when tested as before, gave the same faint evidence of the presence of hydrochloric acid. The residue, when taken from the retort and examined with litmus, was found more acid than before the distillation. It was then carefully evaporated and examined from time to time, with the effect of manifesting a constantly increasing acidity so long as it remained liquid. The heat was then carried still further, so as to dry but not char the material; on moistening it with water it was found even more intensely acid. Heat was again applied and carried to *incipient* charring, and then the material was moistened and tested again, exhibiting a *diminished* acidity. The same experiment repeated and carried to increased charring, showed, on moistening the residue, a still diminishing acidity, and on heating the residue to thorough charring and until all empyreumatic odor ceased to be given off, it was found that *all* acidity had disappeared.

*Ex.* 6.—It was suggested that the acid reaction of the residuum might be due to phosphoric acid, and that it might have been decomposed by the heat employed and the carbon which was present. To determine this, another portion of the same fluid was mixed with three drops of a solution of phosphoric acid, and the mixture was carried through the same series of experiments, giving a successively increasing acidity, with this peculiar result, however—that even after the whole material had been thoroughly charred, as before, and still more highly heated, the acid reaction still remained, thus demonstrating that the acid detected in the product of digestion in the first experiments was *not* the phosphoric.

*Ex.* 7.—To ascertain whether hydrochloric acid, if present in the free state, could resist the distilling heat and remain in the residuum when concentrated, a minute drop was added to a quantity of water so large as to render its reaction undetectible by litmus ; and a like quantity to the fluid of digestion, and both were distilled. In both cases, a *very* distinct evidence of the presence of the acid was obtained in the distillate by a decided precipitate with nitrate of silver.

Experiments 6 and 7 go to show that the acid of the gastric juice, that at least upon which its most decided action depends, is not phosphoric acid, for it does not resist high heat, as that acid is known to do. It is probably not hydrochloric, nor acetic, for these are both highly volatile and are detected readily in the distillate by nitrate of silver.

*Ex.* 8.—This experiment was performed in verification of the doubt just stated, that hydrochloric acid is not present in the free state in the fluid of digestion. A portion of all the digestive fluids obtained from St. Martin, and a quantity vomited at will by another individual, were tested with pure deutoxide of manganese, without giving the slightest trace of the presence of chlorine.

Another portion of the digestive fluids was carefully filtered, and a minute trace of chloride of calcium added to it; the material was then tested with oxalic acid, when immediately the white precipitate of oxalate of lime took place. Had any free hydrochloric acid been present, it would have prevented the appearance of the precipitate by dissolving it. To prove this, another portion of the same gastric fluid was filtered, and a minute quantity of hydrochloric acid and chloride of calcium were added to it. The addition of oxalic acid now produced *no* precipitate. (See Lehmann's Phys. Chem. p. 93, vol. i.)

It thus became a demonstration that the strong acid reaction of these gastric fluids was not due to the presence of free hydrochloric acid. It seems equally clear that it was an *organic* acid, from the fact that it was destroyed by heat, as in Ex. 5 ; and almost certain that it was lactic. To decide this doubt, a portion of the distillate and another of the residue in the retort were tested with zinc, as recommended by Lehmann, (Phys. Chem. p. 92, vol. i.,) with the effect of producing the characteristic crystals of *lactate of zinc.*

It will be remembered that in experiments 4 and 8 a faint evidence of the presence of hydrochloric in the distillate was manifested by the reaction with nitrate of silver. It will also be remembered, that Bernard and Barreswill assert that this hydrochloric acid is due to the decomposition of the alkaline chlorides at a high heat. To ascertain this the following experiment was performed :

*Ex.* 9.—Lactic acid was mixed with chloride of sodium, and the two were heated in a retort. The distillate gave the *faintest* possible trace of opalescence when treated with nitrate of silver. This evidence can scarcely be relied upon, for the solution above described required so high a temperature to produce ebullition, that it was difficult to prevent a spurious distillation of the chloride of sodium along with the vapor, and from this, it is believed, arose the opalescence in the reaction between the distillate and nitrate of silver. If lactic acid can decompose the chloride of sodium, it can only be in very small amount ; *chloride of calcium*, as Lehmann has shown, can be decomposed by lactic acid, and if this be present in gastric juice with lactic acid, we may have hydrochloric acid developed by distillation.

May 8th.—A meal of roast beef, with a small portion of salt as a condiment, was given to St. Martin at 2 P. M. At 3½ o'clock of the same afternoon the contents of the stomach were removed.

The fluid was viscid, inodorous, presented a flocculent deposit and a marked acid reaction; s. g. 1008. The microscope revealed numerous epithelial cells from the mucous membrane of the mouth downwards as far as the stomach, mucous corpuscles, amorphous granular matter, oil globules in great abundance, and transversely striated muscular fibres, in some of which the sarcolemma was softened and ruptured, and the sarcous elements just liberated.

The gastric fluid was carried through the same series of experiments as those to which the product of bread digestion was subjected to, and with a like result. The distillate was distinctly acid, but gave very faint traces of hydrochloric acid. The residuum became most intensely acid as it was concentrated, and the presence of lactic acid was manifested, both in the distillate and the residuum, by the test of the characteristic crystals of lactate of zinc.

From the preceding experiments the following conclusions are fairly deducible:

1st. That the secretions of the stomach when digesting are invariably acid.

2d. That the acid reaction was not due to the presence of phosphoric acid.

3d. That *if* hydrochloric acid was present, it was in very small quantities.

4th. That the main agent in producing the characteristic reaction was *lactic acid.*

It is but just to say that the experiments were conducted with the utmost care and precision, with a single eye to *truth*, and not with a view to support any favorite theory of digestion. So far from this, it may be stated, that the results arrived at are at variance with the doctrines maintained by the writer for many years. Each experiment was repeated several times, so as to leave no room for doubt, and was carefully compared with the results obtained by examination of the fluids discharged at will by another individual.

It is true that this is only one series of observations, and the first that has been published, so far as St. Martin is concerned, since the analyses of Dunglison and Emmett; and now that he has again become a subject for experimentation, perhaps other and different results may be obtained. There may be sources of fallacy, unknown to the writer at the time, which may vitiate the conclusions arrived at; if so, they will be abandoned as readily as those formerly maintained.

Si quid novisti rectius istis, candidus imperti;
Si non, his utere mecum!

(To be continued.)

